# 1-(4-Bromo­phenyl­sulfon­yl)-2-methyl­naphtho­[2,1-*b*]furan

**DOI:** 10.1107/S160053681202939X

**Published:** 2012-07-04

**Authors:** Hong Dae Choi, Pil Ja Seo, Uk Lee

**Affiliations:** a Department of Chemistry, Dongeui University, San 24 Kaya-dong, Busanjin-gu, Busan 614-714, Republic of Korea; b Department of Chemistry, Pukyong National University, 599-1 Daeyeon 3-dong, Nam-gu, Busan 608-737, Republic of Korea

## Abstract

In the title compound, C_19_H_13_BrO_3_S, the 4-bromo­phenyl ring makes a dihedral angle of 64.11 (2)° with the mean plane [r.m.s. deviation = 0.01 (2) Å] of the naphtho­furan ring. In the crystal, mol­ecules are linked by weak C—H⋯O and C—H⋯π inter­actions. The crystal structure also exhibits slipped π–π inter­actions between the central naphtho­furan benzene rings of neighbouring mol­ecules [centroid–centroid distance = 3.559 (2), slippage = 1.036 (2) Å], and between the central naphtho­furan benzene ring and the furan ring of neighbouring mol­ecules [centroid–centroid distance = 3.655 (2), slippage = 1.136 (2) Å].

## Related literature

For background information and the crystal structures of related compounds, see: Choi *et al.* (2008 ▶, 2012 ▶).
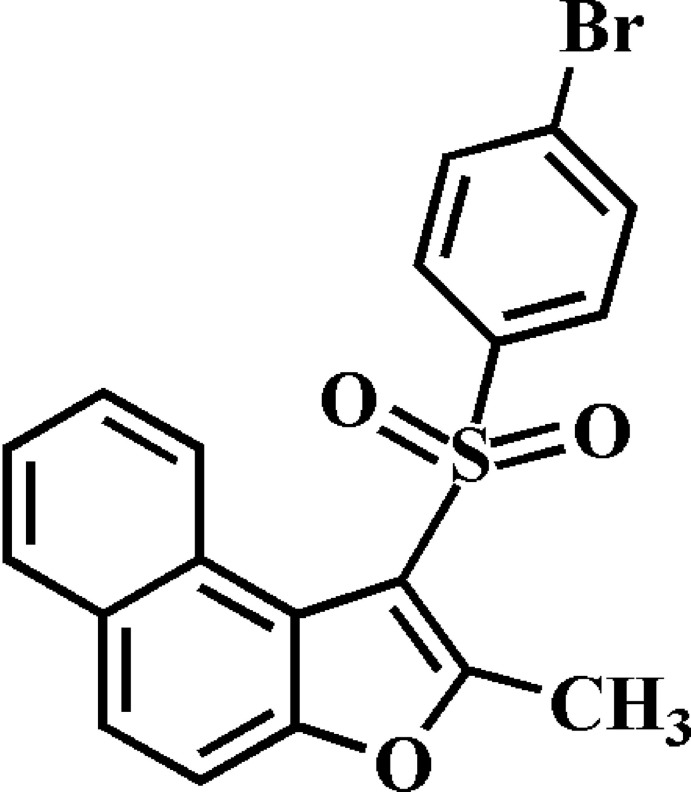


## Experimental

### 

#### Crystal data

C_19_H_13_BrO_3_S
*M* *_r_* = 401.26
Triclinic, 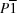

*a* = 6.9579 (2) Å
*b* = 10.9709 (4) Å
*c* = 11.4207 (4) Å
α = 110.510 (2)°
β = 105.886 (2)°
γ = 91.656 (2)°
*V* = 777.77 (5) Å^3^
*Z* = 2
Mo *K*α radiation
μ = 2.79 mm^−1^
*T* = 173 K
0.29 × 0.13 × 0.10 mm

#### Data collection

Bruker SMART APEXII CCD diffractometer
Absorption correction: multi-scan (*SADABS*; Bruker, 2009 ▶) *T*_min_ = 0.555, *T*_max_ = 0.746
14423 measured reflections
3876 independent reflections
3201 reflections with *I* > 2σ(*I*)
*R*_int_ = 0.036

#### Refinement

*R*[*F* ^2^ > 2σ(*F* ^2^)] = 0.032
*wR*(*F* ^2^) = 0.084
*S* = 1.05
3876 reflections
218 parameters
H-atom parameters constrained
Δρ_max_ = 0.49 e Å^−3^
Δρ_min_ = −0.35 e Å^−3^

### 

Data collection: *APEX2* (Bruker, 2009 ▶); cell refinement: *SAINT* (Bruker, 2009 ▶); data reduction: *SAINT*; program(s) used to solve structure: *SHELXS97* (Sheldrick, 2008 ▶); program(s) used to refine structure: *SHELXL97* (Sheldrick, 2008 ▶); molecular graphics: *ORTEP-3* (Farrugia, 1997 ▶) and *DIAMOND* (Brandenburg, 1998 ▶); software used to prepare material for publication: *SHELXL97*.

## Supplementary Material

Crystal structure: contains datablock(s) global, I. DOI: 10.1107/S160053681202939X/ff2074sup1.cif

Structure factors: contains datablock(s) I. DOI: 10.1107/S160053681202939X/ff2074Isup2.hkl

Supplementary material file. DOI: 10.1107/S160053681202939X/ff2074Isup3.cml

Additional supplementary materials:  crystallographic information; 3D view; checkCIF report

## Figures and Tables

**Table 1:** Hydrogen-bond geometry (Å, °) *Cg*1 is the centroid of the C14–C19 4-bromo­phenyl ring.

*D*—H⋯*A*	*D*—H	H⋯*A*	*D*⋯*A*	*D*—H⋯*A*
C16—H16⋯O2^i^	0.95	2.49	3.197 (2)	131
C19—H19⋯O3^ii^	0.95	2.47	3.177 (2)	132
C9—H9⋯*Cg*1^iii^	0.95	2.96	3.782 (2)	120

Symmetry codes: (i) 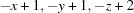; (ii) 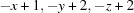; (iii) 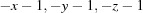.

**Fig. 1.**
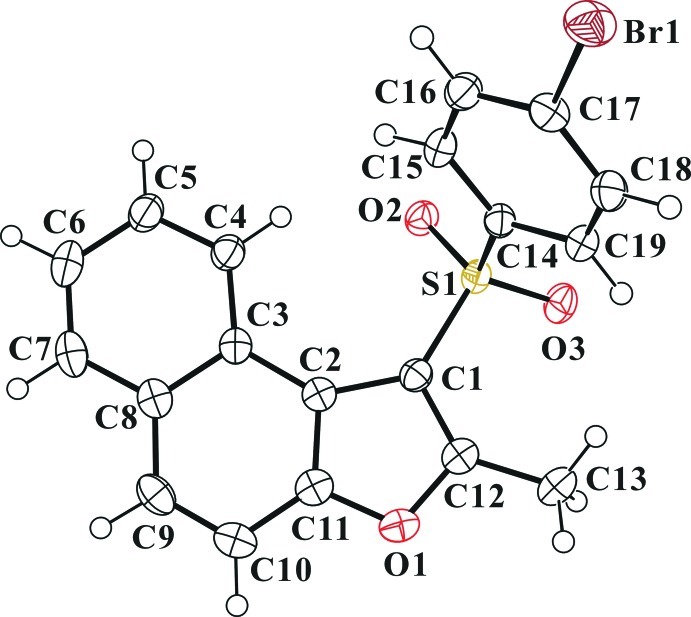
The molecular structure of the title compound with the atom numbering scheme. Displacement ellipsoids are drawn at the 50% probability level. H atoms are presented as small spheres of arbitrary radius.

**Fig. 2.**
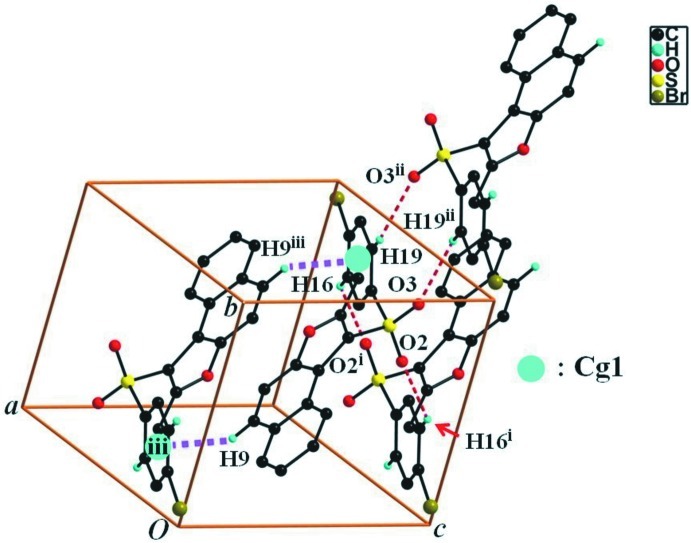
A view of the C—H···O and C—H···π interactions (dotted lines) in the crystal structure of the title compound. H atoms non-participating in hydrogen-bonding were omitted for clarity. [Symmetry codes: (i) - x + 1, - y + 1, - z + 2; (ii) - x + 1, - y + 2, - z + 2; (iii) - x + 1, - y + 1, - z + 1.]

**Fig. 3.**
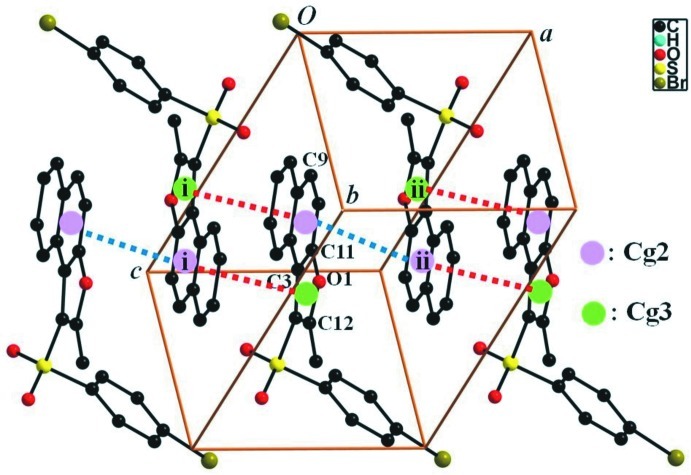
A view of the π–π interactions (dotted lines) in the crystal structure of the title compound. All H atoms were omitted for clarity. [Symmetry codes: (i) - x , - y + 1, - z + 1; (ii)- x + 1, - y + 1, - z + 1.]

## supplementary crystallographic information

### Comment

As a part of our ongoing study of 2-methylnaphtho[2,1-b]furan derivatives containing 1-phenylsulfonyl (Choi *et al.*, 2008) and 1-(4-methylphenylsulfonyl) (Choi *et al.*, 2012) substituents, we report herein the crystal structure of the title compound.

In the title molecule (Fig. 1), the naphthofuran unit is essentially planar, with a mean deviation of 0.017 (2) Å from the least-squares plane defined by the thirteen constituent atoms. The dihedral angle between the 4-bromophenyl ring and the mean plane of the naphthofuran ring is 64.11 (2)°. In the crystal structure (Fig. 2), molecules are connected by weak intermolecular C—H···O and C—H···π interactions (Table 1, Cg1 is the centroid of the C14–C19 4-bromophenyl ring). In the crystal structure (Fig. 3), molecules are connected π–π interactions; the first one between the central naphthofuran benzene rings of neighbouring molecules, with a Cg2···Cg2^ii^ distance of 3.559 (2) Å and an interplanar distance of 3.405 (2) Å resulting in a slippage of 1.036 (2) Å (Cg2 is the centroid of the C2/C3/C8/C9/C10/C11 benzene ring), and the second one between the central naphthofuran benzene ring and the furan ring of neighbouring molecules, with a Cg2···Cg3^i^ distance of 3.655 (2) Å and an interplanar distance of 3.474 (2) Å resulting in a slippage of 1.136 (2) Å (Cg3 is the centroid of the C1/C2/C11/O1/C12 furan ring).

### Experimental

3-Chloroperoxybenzoic acid (77%, 448 mg, 2.0 mmol) was added in small portions to a stirred solution of 1-(4-bromophenylsulfanyl)-2-methylnaphtho [2,1-b]furan (332 mg, 0.9 mmol) in dichloromethane (40 mL) at 273 K. After being stirred at room temperature for 8h, the mixture was washed with saturated sodium bicarbonate solution and the organic layer was separated, dried over magnesium sulfate, filtered and concentrated at reduced pressure. The residue was purified by column chromatography (benzene) to afford the title compound as a colorless solid [yield 71%, m.p. 452–453 K; *R*_f_ = 0.69 (benzene)]. Single crystals suitable for X-ray diffraction were prepared by slow evaporation of a solution of the title compound in acetone at room temperature.

### Refinement

All H atoms were positioned geometrically and refined using a riding model, with C—H = 0.95 Å for aryl and 0.98 Å for methyl H atoms. Uiso(H) = 1.2*U*_eq_(C) for aryl and 1.5*U*_eq_(C) for methyl H atoms. The positions of methyl hydrogens were optimized rotationally.

### Crystal data

**Table:**

C_19_H_13_BrO_3_S	*Z* = 2
*M**_r_* = 401.26	*F*(000) = 404
Triclinic, *P*1	*D*_x_ = 1.713 Mg m^−^^3^
Hall symbol: -P 1	Mo *K*α radiation, λ = 0.71073 Å
*a* = 6.9579 (2) Å	Cell parameters from 6011 reflections
*b* = 10.9709 (4) Å	θ = 2.2–27.9°
*c* = 11.4207 (4) Å	µ = 2.79 mm^−^^1^
α = 110.510 (2)°	*T* = 173 K
β = 105.886 (2)°	Block, colourless
γ = 91.656 (2)°	0.29 × 0.13 × 0.10 mm
*V* = 777.77 (5) Å^3^	

### Data collection

**Table:**

Bruker SMART APEXII CCD diffractometer	3876 independent reflections
Radiation source: rotating anode	3201 reflections with *I* > 2σ(*I*)
Graphite multilayer monochromator	*R*_int_ = 0.036
Detector resolution: 10.0 pixels mm^-1^	θ_max_ = 28.3°, θ_min_ = 2.0°
φ and ω scans	*h* = −9→8
Absorption correction: multi-scan (*SADABS*; Bruker, 2009)	*k* = −14→14
*T*_min_ = 0.555, *T*_max_ = 0.746	*l* = −15→15
14423 measured reflections	

### Refinement

**Table:**

Refinement on *F*^2^	Primary atom site location: structure-invariant direct methods
Least-squares matrix: full	Secondary atom site location: difference Fourier map
*R*[*F*^2^ > 2σ(*F*^2^)] = 0.032	Hydrogen site location: difference Fourier map
*wR*(*F*^2^) = 0.084	H-atom parameters constrained
*S* = 1.05	*w* = 1/[σ^2^(*F*_o_^2^) + (0.0411*P*)^2^ + 0.2709*P*] where *P* = (*F*_o_^2^ + 2*F*_c_^2^)/3
3876 reflections	(Δ/σ)_max_ = 0.001
218 parameters	Δρ_max_ = 0.49 e Å^−^^3^
0 restraints	Δρ_min_ = −0.35 e Å^−^^3^

### Special details

**Table:**

Geometry. All esds (except the esd in the dihedral angle between two l.s. planes) are estimated using the full covariance matrix. The cell esds are taken into account individually in the estimation of esds in distances, angles and torsion angles; correlations between esds in cell parameters are only used when they are defined by crystal symmetry. An approximate (isotropic) treatment of cell esds is used for estimating esds involving l.s. planes.
Refinement. Refinement of F^2^ against ALL reflections. The weighted R-factor wR and goodness of fit S are based on F^2^, conventional R-factors R are based on F, with F set to zero for negative F^2^. The threshold expression of F^2^ > 2sigma(F^2^) is used only for calculating R-factors(gt) etc. and is not relevant to the choice of reflections for refinement. R-factors based on F^2^ are statistically about twice as large as those based on F, and R- factors based on ALL data will be even larger.

### Fractional atomic coordinates and isotropic or equivalent isotropic displacement parameters (Å^2^)

**Table:**

	*x*	*y*	*z*	*U*_iso_*/*U*_eq_	
Br1	1.25056 (3)	0.78521 (2)	1.20765 (2)	0.04071 (10)	
S1	0.33058 (8)	0.71206 (4)	0.86111 (5)	0.02364 (12)	
O1	0.2860 (2)	0.71224 (15)	0.51468 (15)	0.0292 (3)	
O2	0.2305 (2)	0.60540 (13)	0.87692 (14)	0.0279 (3)	
O3	0.2685 (2)	0.83982 (14)	0.90608 (16)	0.0316 (3)	
C1	0.3103 (3)	0.67109 (19)	0.6958 (2)	0.0239 (4)	
C2	0.2740 (3)	0.54502 (19)	0.5872 (2)	0.0230 (4)	
C3	0.2529 (3)	0.40719 (19)	0.5655 (2)	0.0237 (4)	
C4	0.2678 (3)	0.3548 (2)	0.6634 (2)	0.0290 (4)	
H4	0.2924	0.4128	0.7518	0.035*	
C5	0.2476 (4)	0.2215 (2)	0.6336 (2)	0.0337 (5)	
H5	0.2587	0.1886	0.7015	0.040*	
C6	0.2108 (4)	0.1340 (2)	0.5044 (2)	0.0366 (5)	
H6	0.1970	0.0419	0.4844	0.044*	
C7	0.1949 (3)	0.1816 (2)	0.4076 (2)	0.0342 (5)	
H7	0.1694	0.1215	0.3199	0.041*	
C8	0.2153 (3)	0.3179 (2)	0.4338 (2)	0.0270 (4)	
C9	0.1972 (3)	0.3626 (2)	0.3291 (2)	0.0321 (5)	
H9	0.1690	0.3001	0.2422	0.038*	
C10	0.2193 (3)	0.4924 (2)	0.3507 (2)	0.0310 (5)	
H10	0.2082	0.5229	0.2812	0.037*	
C11	0.2592 (3)	0.5787 (2)	0.4802 (2)	0.0270 (4)	
C12	0.3190 (3)	0.7668 (2)	0.6459 (2)	0.0272 (4)	
C13	0.3533 (4)	0.9123 (2)	0.6993 (2)	0.0350 (5)	
H13A	0.4214	0.9430	0.6481	0.053*	
H13B	0.4376	0.9444	0.7911	0.053*	
H13C	0.2234	0.9458	0.6940	0.053*	
C14	0.5890 (3)	0.72911 (18)	0.94600 (19)	0.0234 (4)	
C15	0.6697 (3)	0.62392 (19)	0.9740 (2)	0.0274 (4)	
H15	0.5888	0.5408	0.9405	0.033*	
C16	0.8677 (3)	0.6410 (2)	1.0507 (2)	0.0284 (4)	
H16	0.9231	0.5704	1.0717	0.034*	
C17	0.9844 (3)	0.7619 (2)	1.0966 (2)	0.0280 (4)	
C18	0.9081 (3)	0.8657 (2)	1.0663 (2)	0.0310 (5)	
H18	0.9915	0.9474	1.0965	0.037*	
C19	0.7094 (3)	0.84936 (19)	0.9916 (2)	0.0280 (4)	
H19	0.6548	0.9205	0.9713	0.034*	

### Atomic displacement parameters (Å^2^)

**Table:**

	*U*^11^	*U*^22^	*U*^33^	*U*^12^	*U*^13^	*U*^23^
Br1	0.03136 (14)	0.04733 (16)	0.03750 (15)	−0.00121 (10)	−0.00038 (10)	0.01757 (12)
S1	0.0257 (2)	0.0192 (2)	0.0267 (3)	0.00392 (18)	0.0099 (2)	0.00780 (19)
O1	0.0271 (8)	0.0331 (8)	0.0317 (8)	0.0034 (6)	0.0083 (7)	0.0178 (7)
O2	0.0308 (8)	0.0245 (7)	0.0309 (8)	0.0017 (6)	0.0122 (7)	0.0113 (6)
O3	0.0346 (8)	0.0241 (7)	0.0386 (9)	0.0103 (6)	0.0165 (7)	0.0102 (6)
C1	0.0223 (10)	0.0236 (9)	0.0261 (10)	0.0041 (8)	0.0067 (8)	0.0100 (8)
C2	0.0161 (9)	0.0265 (9)	0.0263 (10)	0.0037 (7)	0.0062 (8)	0.0101 (8)
C3	0.0161 (9)	0.0245 (9)	0.0269 (10)	0.0035 (7)	0.0041 (8)	0.0072 (8)
C4	0.0297 (11)	0.0254 (10)	0.0304 (11)	0.0052 (8)	0.0068 (9)	0.0100 (9)
C5	0.0369 (12)	0.0254 (10)	0.0367 (13)	0.0050 (9)	0.0075 (10)	0.0119 (10)
C6	0.0354 (12)	0.0223 (10)	0.0458 (14)	0.0048 (9)	0.0109 (11)	0.0064 (10)
C7	0.0295 (11)	0.0288 (11)	0.0328 (12)	0.0015 (9)	0.0077 (10)	−0.0007 (9)
C8	0.0176 (9)	0.0305 (10)	0.0292 (11)	0.0035 (8)	0.0067 (8)	0.0070 (9)
C9	0.0243 (10)	0.0408 (12)	0.0245 (11)	0.0016 (9)	0.0066 (9)	0.0050 (9)
C10	0.0241 (10)	0.0431 (12)	0.0275 (11)	0.0044 (9)	0.0077 (9)	0.0153 (10)
C11	0.0190 (9)	0.0314 (10)	0.0325 (11)	0.0056 (8)	0.0079 (8)	0.0139 (9)
C12	0.0233 (10)	0.0285 (10)	0.0319 (11)	0.0046 (8)	0.0088 (9)	0.0134 (9)
C13	0.0399 (13)	0.0276 (11)	0.0429 (14)	0.0050 (9)	0.0135 (11)	0.0186 (10)
C14	0.0263 (10)	0.0204 (9)	0.0231 (10)	0.0033 (7)	0.0085 (8)	0.0068 (8)
C15	0.0298 (11)	0.0200 (9)	0.0318 (11)	0.0026 (8)	0.0103 (9)	0.0084 (8)
C16	0.0311 (11)	0.0257 (10)	0.0304 (11)	0.0060 (8)	0.0101 (9)	0.0120 (9)
C17	0.0263 (10)	0.0319 (11)	0.0250 (11)	0.0020 (8)	0.0076 (9)	0.0100 (9)
C18	0.0335 (11)	0.0259 (10)	0.0287 (11)	−0.0037 (9)	0.0056 (9)	0.0079 (9)
C19	0.0344 (11)	0.0207 (9)	0.0278 (11)	0.0032 (8)	0.0080 (9)	0.0091 (8)

### Geometric parameters (Å, º)

**Table:**

Br1—C17	1.888 (2)	C7—H7	0.9500
S1—O2	1.4340 (14)	C8—C9	1.421 (3)
S1—O3	1.4376 (15)	C9—C10	1.354 (3)
S1—C1	1.744 (2)	C9—H9	0.9500
S1—C14	1.763 (2)	C10—C11	1.393 (3)
O1—C12	1.353 (3)	C10—H10	0.9500
O1—C11	1.370 (2)	C12—C13	1.482 (3)
C1—C12	1.366 (3)	C13—H13A	0.9800
C1—C2	1.457 (3)	C13—H13B	0.9800
C2—C11	1.375 (3)	C13—H13C	0.9800
C2—C3	1.440 (3)	C14—C19	1.389 (3)
C3—C4	1.407 (3)	C14—C15	1.396 (3)
C3—C8	1.424 (3)	C15—C16	1.382 (3)
C4—C5	1.374 (3)	C15—H15	0.9500
C4—H4	0.9500	C16—C17	1.383 (3)
C5—C6	1.396 (3)	C16—H16	0.9500
C5—H5	0.9500	C17—C18	1.381 (3)
C6—C7	1.360 (3)	C18—C19	1.380 (3)
C6—H6	0.9500	C18—H18	0.9500
C7—C8	1.413 (3)	C19—H19	0.9500
			
O2—S1—O3	118.77 (9)	C9—C10—C11	116.5 (2)
O2—S1—C1	109.36 (9)	C9—C10—H10	121.8
O3—S1—C1	107.02 (9)	C11—C10—H10	121.8
O2—S1—C14	107.25 (9)	O1—C11—C2	111.59 (18)
O3—S1—C14	106.85 (9)	O1—C11—C10	121.95 (19)
C1—S1—C14	107.03 (9)	C2—C11—C10	126.4 (2)
C12—O1—C11	107.05 (16)	O1—C12—C1	110.30 (18)
C12—C1—C2	107.19 (18)	O1—C12—C13	113.74 (18)
C12—C1—S1	120.68 (16)	C1—C12—C13	136.0 (2)
C2—C1—S1	132.06 (15)	C12—C13—H13A	109.5
C11—C2—C3	117.54 (19)	C12—C13—H13B	109.5
C11—C2—C1	103.83 (17)	H13A—C13—H13B	109.5
C3—C2—C1	138.62 (19)	C12—C13—H13C	109.5
C4—C3—C8	118.08 (19)	H13A—C13—H13C	109.5
C4—C3—C2	125.39 (19)	H13B—C13—H13C	109.5
C8—C3—C2	116.52 (19)	C19—C14—C15	120.02 (19)
C5—C4—C3	121.3 (2)	C19—C14—S1	119.99 (16)
C5—C4—H4	119.4	C15—C14—S1	119.88 (15)
C3—C4—H4	119.4	C16—C15—C14	119.82 (18)
C4—C5—C6	120.6 (2)	C16—C15—H15	120.1
C4—C5—H5	119.7	C14—C15—H15	120.1
C6—C5—H5	119.7	C15—C16—C17	119.3 (2)
C7—C6—C5	119.4 (2)	C15—C16—H16	120.4
C7—C6—H6	120.3	C17—C16—H16	120.4
C5—C6—H6	120.3	C18—C17—C16	121.5 (2)
C6—C7—C8	121.9 (2)	C18—C17—Br1	119.98 (16)
C6—C7—H7	119.1	C16—C17—Br1	118.51 (17)
C8—C7—H7	119.1	C19—C18—C17	119.25 (19)
C7—C8—C9	119.7 (2)	C19—C18—H18	120.4
C7—C8—C3	118.7 (2)	C17—C18—H18	120.4
C9—C8—C3	121.7 (2)	C18—C19—C14	120.12 (19)
C10—C9—C8	121.3 (2)	C18—C19—H19	119.9
C10—C9—H9	119.3	C14—C19—H19	119.9
C8—C9—H9	119.3		
			
O2—S1—C1—C12	154.18 (16)	C12—O1—C11—C10	179.18 (19)
O3—S1—C1—C12	24.32 (19)	C3—C2—C11—O1	−178.77 (16)
C14—S1—C1—C12	−89.95 (18)	C1—C2—C11—O1	0.9 (2)
O2—S1—C1—C2	−22.4 (2)	C3—C2—C11—C10	2.2 (3)
O3—S1—C1—C2	−152.26 (19)	C1—C2—C11—C10	−178.2 (2)
C14—S1—C1—C2	93.5 (2)	C9—C10—C11—O1	179.53 (18)
C12—C1—C2—C11	−1.5 (2)	C9—C10—C11—C2	−1.5 (3)
S1—C1—C2—C11	175.45 (16)	C11—O1—C12—C1	−1.1 (2)
C12—C1—C2—C3	178.0 (2)	C11—O1—C12—C13	179.40 (17)
S1—C1—C2—C3	−5.0 (4)	C2—C1—C12—O1	1.6 (2)
C11—C2—C3—C4	178.58 (19)	S1—C1—C12—O1	−175.75 (14)
C1—C2—C3—C4	−0.9 (4)	C2—C1—C12—C13	−179.0 (2)
C11—C2—C3—C8	−0.9 (3)	S1—C1—C12—C13	3.7 (4)
C1—C2—C3—C8	179.6 (2)	O2—S1—C14—C19	−155.16 (16)
C8—C3—C4—C5	0.2 (3)	O3—S1—C14—C19	−26.80 (19)
C2—C3—C4—C5	−179.3 (2)	C1—S1—C14—C19	87.57 (18)
C3—C4—C5—C6	−0.2 (4)	O2—S1—C14—C15	21.08 (19)
C4—C5—C6—C7	0.0 (4)	O3—S1—C14—C15	149.44 (16)
C5—C6—C7—C8	0.2 (3)	C1—S1—C14—C15	−96.18 (17)
C6—C7—C8—C9	−179.9 (2)	C19—C14—C15—C16	2.1 (3)
C6—C7—C8—C3	−0.2 (3)	S1—C14—C15—C16	−174.17 (15)
C4—C3—C8—C7	0.0 (3)	C14—C15—C16—C17	−1.2 (3)
C2—C3—C8—C7	179.52 (18)	C15—C16—C17—C18	−0.7 (3)
C4—C3—C8—C9	179.72 (19)	C15—C16—C17—Br1	177.18 (15)
C2—C3—C8—C9	−0.7 (3)	C16—C17—C18—C19	1.8 (3)
C7—C8—C9—C10	−178.8 (2)	Br1—C17—C18—C19	−176.05 (16)
C3—C8—C9—C10	1.4 (3)	C17—C18—C19—C14	−1.0 (3)
C8—C9—C10—C11	−0.4 (3)	C15—C14—C19—C18	−1.0 (3)
C12—O1—C11—C2	0.1 (2)	S1—C14—C19—C18	175.27 (16)

### Hydrogen-bond geometry (Å, º)

Cg1 is the centroid of the C14–C19 4-bromophenyl ring.

**Table:**

*D*—H···*A*	*D*—H	H···*A*	*D*···*A*	*D*—H···*A*
C16—H16···O2^i^	0.95	2.49	3.197 (2)	131
C19—H19···O3^ii^	0.95	2.47	3.177 (2)	132
C9—H9···*Cg*1^iii^	0.95	2.96	3.782 (2)	120

Symmetry codes: (i) −*x*+1, −*y*+1, −*z*+2; (ii) −*x*+1, −*y*+2, −*z*+2; (iii) −*x*−1, −*y*−1, −*z*−1.
